# A Vehicle Active Safety Model: Vehicle Speed Control Based on Driver Vigilance Detection Using Wearable EEG and Sparse Representation

**DOI:** 10.3390/s16020242

**Published:** 2016-02-19

**Authors:** Zutao Zhang, Dianyuan Luo, Yagubov Rasim, Yanjun Li, Guanjun Meng, Jian Xu, Chunbai Wang

**Affiliations:** 1 School of Mechanical Engineering, Southwest Jiaotong University, Chengdu 610031, China; meng_guanjun@my.swjtu.edu.cn; 2 School of Information Science & Technical, Southwest Jiaotong University, Chengdu 610031, China; luodianyuan@my.swjtu.edu.cn (D.L.); rasim_yagubov@hotmail.com (Y.R.); liyanjun@my.swjtu.edu.cn (Y.L.); 3 The Psychological Research and Counseling Center, Southwest Jiaotong University, Chengdu 610031, China; xujianlm@gmail.com; 4 The Department of Industrial & Manufacturing Systems Engineering, Iowa State University, Ames, IA 50011, USA; chbwang@iastate.edu

**Keywords:** wearable electroencephalographic, vigilance detection, vehicle active safety, vehicle speed control, sparse representation, brain-computer interface

## Abstract

In this paper, we present a vehicle active safety model for vehicle speed control based on driver vigilance detection using low-cost, comfortable, wearable electroencephalographic (EEG) sensors and sparse representation. The proposed system consists of three main steps, namely wireless wearable EEG collection, driver vigilance detection, and vehicle speed control strategy. First of all, a homemade low-cost comfortable wearable brain-computer interface (BCI) system with eight channels is designed for collecting the driver’s EEG signal. Second, wavelet de-noising and down-sample algorithms are utilized to enhance the quality of EEG data, and Fast Fourier Transformation (FFT) is adopted to extract the EEG power spectrum density (PSD). In this step, sparse representation classification combined with k-singular value decomposition (KSVD) is firstly introduced in PSD to estimate the driver’s vigilance level . Finally, a novel safety strategy of vehicle speed control, which controls the electronic throttle opening and automatic braking after driver fatigue detection using the above method, is presented to avoid serious collisions and traffic accidents. The simulation and practical testing results demonstrate the feasibility of the vehicle active safety model.

## 1. Introduction

A growing number of traffic accidents have become a serious social safety problem in recent years. One of the main factors has been the obvious drop in the driver’s perceptual senses, such as feeling, recognition, and automobile control abilities, as they feel sleepy. Statistics show that the leading cause of fatality and injury in traffic accidents is the driver’s diminished vigilance level [1,2]. Analysis of literature suggests that driver vigilance contributes to approximately 43% of vehicle crashes and 27% of near crashes [3]. Fatal crashes reported from Washington D.C. indicate that driver fatigue caused a large proportion of crashes (*i.e.*, 10% in 2005 and 16% in 2009, for total 5474 fatigue-related fatalities in 2009) [4]. In Europe, statistics report that about 10% to 20% of all traffic accidents are caused by driver’s fatigue due to a diminished vigilance level. The National Sleep Foundation (NSF) reported that 51% of Americans have driving while feeling drowsy and 17% admit that they had actually fallen asleep [5].

Technologies such as pretension seat belt, airbag, antilock brake system, traction control system, and electronic stability programs, can protect people in collisions only to a certain extent. It is important to develop systems that actively detect a driver’s level of vigilance and control the vehicle speed when he is driving fatigued. Over the past several decades, many researchers have focused on driver’s vigilance detection. They solved this problem by sending a warning to driver when the driver appears drowsy. Nevertheless, these technologies are not helpful in the control of vehicle because the majority of drivers believe that they are able to control the vehicle. However, they actually are not. With increasing vehicle speed, the dynamic vision, vision of the driver in motion, and dynamic vision field (vision field of the driver in motion) decrease. Generally, dynamic vision is 10%–20% lower than a static vision. For instance, when vehicle speed is 60 km/h, the driver can see traffic signs within 200 m. When vehicle speed is up to 80 km/h, the driver can only see traffic signs within 160 m ahead. Therefore, it is necessary and efficient to control the vehicle speed or decelerate to prevent a collision when the driver is drowsy.

### 1.1. Driver Vigilance Detection Technologies

Currently, technologies of detecting driver vigilance are developing rapidly. The most popular approaches are classified into three categories [6,7,8]. The first category focuses on the movements of the vehicle [9], such as detecting the lane departure, steering wheel movement, the pressure of driving pedal. If the movement of the vehicle is abnormal, the driver is regarded as drowsy. Although this technology provides a noninvasive way for correcting the driver, it is difficult to construct a common model for drowsy driving due to variability of an in dividual’s driving behavior and changes of road circumstances.

The second category analyzes the changes of driver’s physical behaviors [10,11,12,13,14,15], such as eye tracking, yawning, percent eye closure (PERCLOS), blink frequency, nodding frequency, face position, and the inclination of the driver’s head. In literature [10,11,12,13,14,15], measuring eyelid movement, face expression and head pose using video cameras are effective ways o driver vigilance detection based on machine vision and computer hardware technologies. The use of multiple visual parameters and the information fusion of various driver facial visual cues, were used to model driver vigilance and to predict driver fatigue [14,15]. Usually, in such an algorithm, a video is used to analyze and classify the vigilance level of the driver. Video is susceptible to environmental and driving conditions such as light conditions, glasses worn by the driver, and so on. Furthermore, false estimation can also be caused by variability of the driver’s behavior, such as sleepiness with eyes open.

The last category is the physiological signal [16,17,18,19,20,21,22,23,24,25,26,27,28] for driver drowsiness detection, using electrocardiosignal (ECG), electrooculographic (EOG), electroencephalographic (EEG), and heart rate variability (HRV). These systems are more reliable because physiological drowsiness signs are well known and rather similar from one driver to another. EEG is always regarded as a “gold standard” of vigilance detection. In [16], Šušmáková described the relationship between human sleepiness and EEG. It indicated that some existent rhythm components, theta (4 Hz–8 Hz), alpha (8 Hz–14 Hz), and beta (14 Hz–34 Hz) in EEG had a close relationship with the driver’s vigilance levels. There is a positive correlation between the power of theta or alpha rhythm and drowsiness, and a negative correlation between the power of beta rhythm and being awake. In this category, the first and most important part is the acquisition of EEG of driver. However, the traditional laboratory equipment is too large and troublesome to use. To extend the application of EEG to the drowsy driving field, many researchers began to develop portable equipment for EEG collection. Lin *et al.* have designed a series of wearable BCI systems in [17,18] to detect driver vigilance. Their system possesses three functions. The first function is EEG acquisition and amplifying, and the second function is data transmission implemented in CPLD (complex programmable logic device), *etc.* The third function is vigilance detection implemented in OMAP (open multimedia architecture platform), *etc.* In literature [19,20,21], Rodger *et al.* had also made significant improvement of interface neuro-physiological behavior performance over existing techniques, which authors proposed a NeuroIS knowledge discovery approach, a study on emotion and memory, improving memory, and software training results for BCI systems. Independent component analysis (ICA), wavelet, and filters are usually used in interference removal. Next, a vigilance detection algorithm is implemented to distinguish different states of the driver [4,22,23,24,25,26,27]. In [4], the author explores many experimental results to verify the relationship between EEG power spectrum density (PSD) and drowsiness. The power of alpha and beta rhythm in an alert state is greater than in a drowsy state. The power of the theta rhythm in an alert state is lower than in a drowsy state. In [22], Jung *et al.* proposed a model of estimating alertness based on EEG power spectrum as early as 1997. In their paper, principal component analysis (PCA) is used for EEG feature extraction, and artificial neural networks (ANN) are used to establish an alertness model. The results show that continuous, accurate, noninvasive, and nearly real-time estimation of vigilance levels using EEG is feasible. In [23], as a kind of EEG feature, power spectrum density is extracted to construct a drowsy model for vigilance classification. Yu *et al.* use continuous wavelet transform (CWT) to extract the rhythm features of EEG and use sparse representation classification to accomplish the classification task in [24]. To enhance the performance of sparse representation classification, k-singular value decomposition (KSVD) proposed by Aharon, *et al.* in [25] is explores. A multi-channel EEG signal model during a working memory task was presented in literature [26]. In [27], a mobile healthcare device for dirver vigilance detection using wavelet-based EEG and respiration signals was presented. The driver’s health condition was analyzed from evaluating the heart rate variability in the time and frequency domains. In [28], a new evaluation model of driver fatigue is established with integration of four fatigue-based indicators with a dynamic Bayesian network (DBN). The results show that it is more accurate to evaluate driver fatigue than the sole EEG-based indicator. The difficulties of these physiological signal measures as on-road driver vigilance detection monitors are in how to obtain EEG recordings comfortably under driving conditions and classify the driver drowsiness with so many EEG signals. Nevertheless, the physiological signal measures are believed accurate, valid, and objective in determining driver vigilance.

### 1.2. Vehicle Speed Control Algorithms after Driver Vigilance 

After the driver is drowsy, some proposed systems give warnings to the driver in order to avoid traffic accidents [9,15,17]. Despite warning of fatigue driving, most drivers believe they can drive safely. Under the circumstances when the driver’s response and vigilance continue to slow down, the vehicle active safety strategy is an important optional system that provides speed control in order to prevent traffic collisions. Adaptive cruise control (ACC) and stop-and-go strategies are related to vehicle speed control. The former is mainly involved in the inter-distance control on the road where the automoble drives at a constant speed, whereas the latter deals with vehicle commuting in municipal areas with frequent stops, decelerations, and accelerations [29]. In [30], Li proposed an active control strategy to keep vehicles away from possible collisions owing to distracted driving or drivers’ attention. In [31], the literature examines drivers’ adaptation using a conceptual model of adaptive behavior. In [32], Zhang *et al.* present a reversing speed control for vehicle safety. The final simulation and experimants show the validity of the vehicle reversing speed control. McCall *et al.* proposed a novel safety system to reduce rear-end collisions based on predictive braking assistance in [33]. In [34], Keller *et al.* present a active pedestrian safety system that fuses sensors, state analysis, decision support, and autombile control. In [35,36], Naranjo *et al.* proposed an ACC system that was used for vehicle safety. The related work treats the vehicle speed control by environment perception [37], road condition detection [38,39], and driver active state detection [40].

Despite the success of the existing approaches/systems for driver vigilance detection and vehicle speed control, a variety of factors still challenge researchers. Much research has been conducted on driver vigilance detection systems, focusing on the following three main problems: (1) how to find a higher-reliability and lower-cost comfortable wearable EEG system that are currently widely used to get the EEG signal of driver; (2) how to detect and recognize the driver fatigue from so much data; and (3) how to apply the vehicle speed control algorithm after driver fatigue detection using the above method in preventing collisions. Until now, few have investigated deeply and systematically vehicle active safety technology based on vigilance detection using wireless wearable EEG signals. The existent lane departure warning system and vehicle collision warning system are established on the driving state and environment to avoid collision. It is imperative to take measures to reduce collisions based on the study of the driver’s vigilance state using EEG signals. In this paper, we introduce an active safety method for vehicle speed control based on driver vigilance detection using wearable EEG and sparse representation. A homemade eight-channel low-cost comfortable wearable brain–computer interface (BCI) hardware system is developed to collect the EEG signal of driver. We can transmit the data of BCI hardware to a personal computer (PC)/field-programmable gate array (FPGA)/digital signal processor (DSP) via a Bluetooth interface. Then wavelet de-noising and down-sample algorithm are utilized to enhance the quality of EEG data and Fast Fourier Transformation (FFT) is adopted to extract the EEG power spectrum density (PSD). Sparse representation classification combined with KSVD is, firstly, implemented in PSD to estimate the driver vigilance level. After driver vigilance detection and recognition, a novel vehicle speed control strategy will make a decision to decelerate or brake. The results of the practical test and simulation show the feasibility of the proposed vehicle active safety model.

The rest of this paper is organized as follows. In Section 2, the general system architecture is presented. Section 3 focuses on low-cost wearable BCI system for EEG collection using our homemade eight-channel BCI system. Sparse representation classification for vigilance detection is described in Section 4, and vehicle speed control strategy is proposed in Section 5. The system simulation and validation are reported in Section 6. Finally, some conclusions are given in Section 7.

## 2. System Architecture

**Figure 1:**
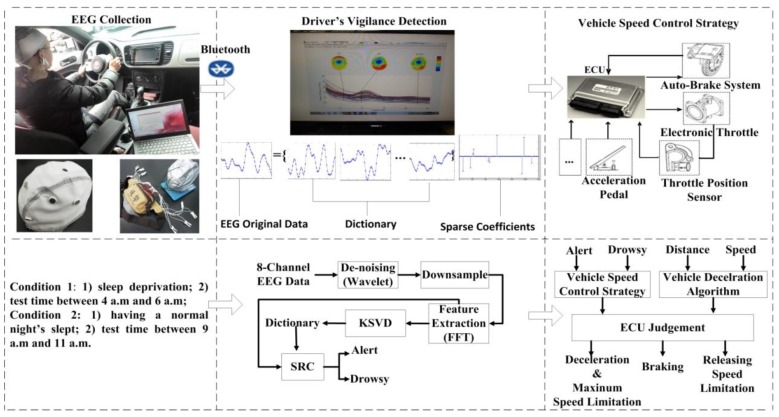
Flowchart of the proposed vehicle active safety model.

The general architecture of our system shown in Figure 1, includes three major steps: (1) a wearable BCI system for EEG collection; (2) driver vigilance detection using sparse representation classification combined with KSVD; and (3) a vehicle speed control strategy.

In the first step, when a driver is driving, a homemade eight-channel wearable BCI system is used to collection the EEG signals of the driver and then transmits its recorded data to PC/FPGA/DSP via a Bluetooth interface. Our BCI system consists of eight stainless steel dry electrodes. It incorporates the use of a wearable EEG device to record EEG signals from the head region of the driver. To extend the application of EEG to the drowsy driving field, the portable system is developed for EEG collection with all of the chips (including Bluetooth model and batteries) in a small bag. In order to acquire the data from different vigilance levels, we set the experimental conditions as the left of Figure 1. In this paper, we define two vigilance levels: alert and drowsy. In our experiment and simulation, we use a PC to process simulation data. The improved versions will be completed in the later experiment using DSP or FPGA.

The second step is driver’s vigilance detection using sparse representation classification combined with KSVD. As shown in the middle block of Figure 1, after the EEG original data is collected using our eight-channel wearable BCI equipment, de-noising is implemented to remove some interference using wavelets. Then the data is down-sampled to 128 Hz in order to reduce the computation load. PSD is extracted as a feature of each state using FFT. Sparse representation classification combined with KSVD is implemented in PSD to estimate the driver’s vigilance level, and the dictionary is prepared before classification and is trained using KSVD.

The final step is the vehicle speed control strategy in the right block of Figure 1. Despite the success of the existing approaches/systems for driver vigilance detection and ACC system, there are few studies about vehicle active safety technology based on vigilance detection that are deeply and systematically based on vigilance detection using wireless wearable EEG signals. In this paper, an active safety strategy for vehicle speed control based on vigilance detection using EEG and sparse representation is developed.

After driver vigilance detection, a vehicle speed control strategy determines what steps it takes to control the speed of vehicle as shown in Figure 1. According to the following car speed and distance detected by a binocular camera system, the vehicle deceleration model gives a safe deceleration method in the course of speed control. After the driver continues drowsy driving, the electronic control unit (ECU) receives the driver vigilance detection information from the second step and automatically controls vehicle speed. When the driver is detected in deep drowsiness, the ECU will control the throttle opening and auto brake to make the car decelerate or brak slowly to reduce the accident rate.

## 3. Wearable BCI System for EEG Collection

**Figure 2:**
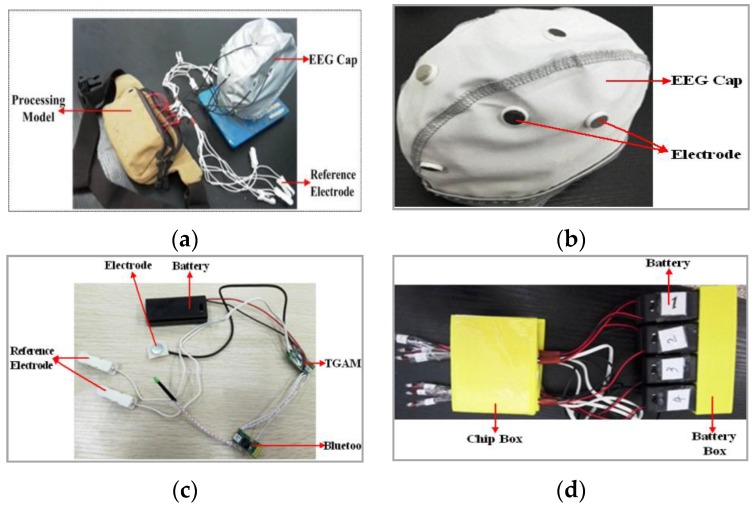
Fabrication of our proposed wearable BCI system. (**a**) Wearable BCI system; (**b**) Electrode cap; (**c**) Single-channel wearable EEG collection module; (**d**) The processing module.

EEG data is very important to our driver vigilance detection. The basic scheme of our EEG-based BCI system is proposed as in Figure 2. The wearable BCI system consists of EEG cap, reference electrodes, and a processing model as shown in Figure 2a. In Figure 2b, the positions of eight dry electrodes installed in EEG cap are corresponding to the cerebrum areas of O1, O2, C3, Cz, C4, P3, Pz, and P4. The installation positions on the head have a close relationship with driver vigilance level detection. In our system, the wearable EEG cap is suitable to comfortably collect the driver’s brain signal data. It has eight single-channel EEG collection modules. As shown in Figure 2c for a single module, the structure has a five-part structure: (1) a stainless steel dry electrode; (2) TGAM module (the chip used to process EEG signal); (3) Bluetooth module; (4) reference electrode, and (5) battery module. The EEG signal is obtained by the stainless steel dry electrodes firstly, and then amplify and filter by the ThinkGear asic module (TGAM) model with the hardware filtering 3 Hz to 100 Hz and sampling rate of 512 Hz. Next, the EEG signal is transmitted to our PC via Bluetooth. The reference electrode provides reference potential for our stainless steel dry electrode. The system is powered by eight 3-V DC batteries. Figure 2d shows the processing module, including TGMA, Bluetooth, and batteries installed into some boxes, which are designed by *SolidWorks* software and manufactured by a 3D printer. At last, all chips of TGAM, Bluetooth, and batteries are arranged into a bag shown in Figure 2a to make our equipment more wearable. Although there are many commercial solutions, in our paper we want to design a low-cost wearable EEG system for our research. The cost of our homemade wearable EEG system is very cheap. Although it is not the cheapest, we think our homemade wearable EEG system has cost advantages compared with some of the commercial solutions. At the same time, the homemade wireless wearable EEG system is necessary for our future research. Our research is vehicle safety and the speed control module needs some interface via EEG. Our homemade wearable EEG system can meet our requirements.

**Figure 3:**
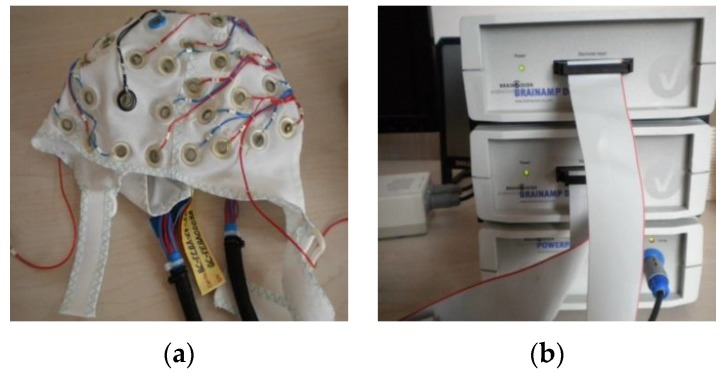
BP equipment in our previous study for comparison. (**a**) BrainCap; (**b**) BrainAmp.

To improve the efficiency of our homemade wearable BCI system in data acquisition accuracy, a 64-channel EEG provided by BRAIN PRODUCTS (BP) was used in our previous study and analysis, in the course of which we have a clear understanding about drowsiness-related EEG signals. As shown in Figure 3, the commercial BP system consists of BrainCap, BrainAmp, and the Recorder Analysis Software. In Figure 3a, BrainCap is an EEG acquisition cap with 64 channels and high-quality Ag/AgCl sensors. BrainAmp is used to amplify the EEG signal in Figure 3b. Recorder is software used to view, accept, reserve, and process EEG signals.

In this paper, we acquire EEG signal from both equipments. We called the signal set **Set**_ijk_, where i = {BP, wearable homemade BCI system (HBS)} indicates the type of apparatus, j = {*a*, *d*} shows the state of subject (*a* is the state of alert, *d* is drowsy), and k = {O1, O2, C3, Cz, C4, P3, Pz, P4}, representing the number of channels.

## 4. Driver Vigilance Detection

After we get the EEG original signal, data processing is processed using MATLAB R2012a in three main steps described as follows.

### 4.1. Data Preprocessing

**Figure 4:**
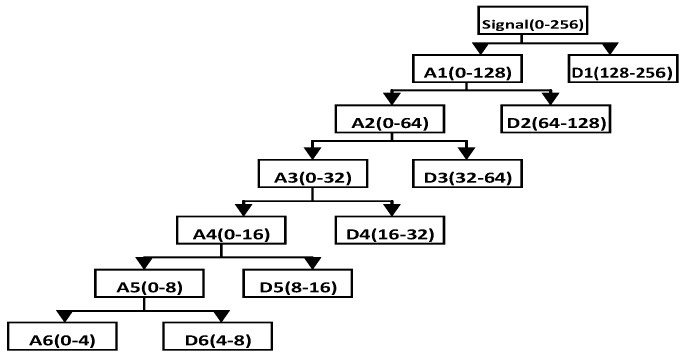
Decomposition space tree and frequency range of wavelet transform.

This process contains de-noising and downsampling. Discrete wavelet transformation (DWT) is an excellent time-frequency analysis tool in various fields of signal processing, for example, for de-noising of EEG. In this paper, we explore the application of the wavelet de-noising method for EEG signals according to its multi-resolution for the non-stationary EEG signal. A six-layer decomposition of db5 wavelet is implemented in the original EEG signal to get the sub-bands’ wavelet detail coefficients (D_i_, i = 1, 2, 3, 4, 5, 6) and approximation coefficients (A_i_, i = 1, 2, 3, 4, 5, 6). The decomposition space tree and frequency range are shown in Figure 4. The range of EEG frequency is from 0.5 Hz to 50 Hz. By reconstructing the decomposition coefficients of d3, d4, d5, d6, we can extract the useful EEG signal and remove some low frequency and high frequency interference, as mentioned above.

In addition, **Set**_HBS_ is downsampled to a sampling rate of 128Hz and **Set**_BP_ is downsampled to a sampling rate of 100 Hz to reduce the computation load.

### 4.2. Feature Extraction

**Figure 5:**
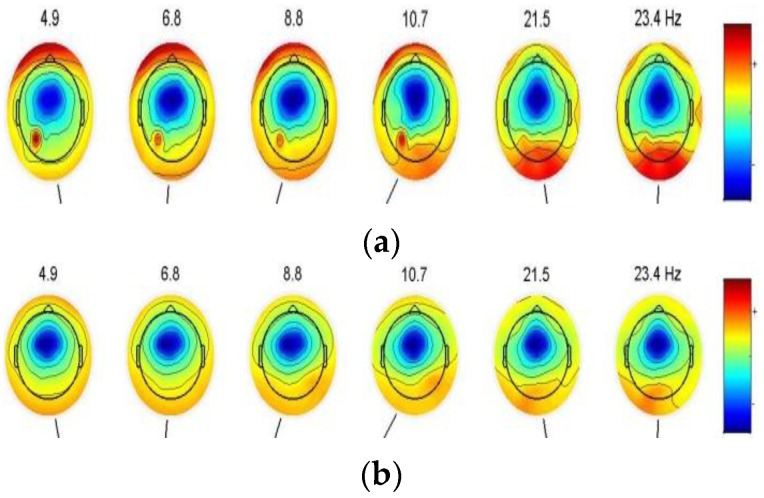
Power scalp topographies of some frequency components. (**a**) Power scalp topographies of the alert state; (**b**) Power scalp topographies of the drowsy state.

It is very important to know the relationship between EEG and drowsiness for feature extraction. Firstly, **Set**_BP_ is analyzed using eeglab v12.0.0.0b (toolbook) of *MATLAB* to reveal the features of the EEG. Figure 5 shows the power scalp topographies of some frequency components in the states of (a) alert and (b) drowsy.

In Figure 5, we can observe many significant differences of the frequency distribution on the scalp. In the alert case, the more low frequency components are found in the area of the forehead, the more high-frequency components are distributed in the area of occipital. In the case of drowsiness, low-frequency components and high-frequency components in the area of the forehead and occipital region are approximately uniform. We can distinguish between different states from two areas of occipital and forehead using power spectral density of different frequency components. Since the signal in the forehead area is susceptible to the eye movement artifacts [18], the PSD of the occipital area (O1, O2) is adopted to distinguish between the two states. Then, the feature of the EEG signal at time *t* was the average PSD of the previous *s* s and *t* s. The PSD of each second is calculated using a 128-point FFT and then converted into a logarithmic scale. Next, a Rectangle Window is used to extract PSD of *theta*, *alpha*, *beta* rhythms, reported as the significant index for the driving error. At last, we stack the feature into a feature set **F**.

### 4.3. Driver Vigilance Detection Based on Sparse Representation Classification Combined with KSVD

Driver vigilance detection is the most important part in our vehicle active safety model. The sparse representation classification algorithm, one of the most popular classifiers used in pattern recognition in recent years, is used for the problem of vigilance classification. Moreover, KSVD is utilized to learn an over-complete dictionary for each level of the vigilance state. Meanwhile, L_0_ minimization is used in solving the sparse representation problem. A minimal residual method is engaged to solve the classification problem for driver vigilance detection. In this paper, the sparse representation classification combined with KSVD is firstly introduced to implement in PSD to estimate the driver vigilance level. Although other algorithms have been used previously for the same task, we want to try to construct a novel algorithm for driver vigilance detection using sparse representation classification and KSVD.

#### 4.3.1. Sparse Representation

In a sparse representation model, an EEG feature **y** (**y** ∈ *R^p^*) belonging to the set of **F***_ijk_*, can be represented as a linear combination of atoms from an over-complete dictionary **DIC** = [**d***_1_*, **d***_2_*, …, **d***_w_*] (**DIC**∈***R****^p×w^*, *w* > *p*), as Equation (1), where **d***_w_* is the atom of the dictionary, *w* is the number of atoms, and *p* is the length of **d***_w_*:

**y** = **d***_1_α_1_* + **d***_2_α_2_* + … + **d***_w_α_w_* = **DIC***α* (1)

where *α* = [*α_1_, α_2_, …, α_w_*] is the sparse coefficient matrix, which only in a small fraction are non-zero. The optimum solution of sparse coefficients can be formulated as Equation (2), which denotes a L_0_ minimization problem:

(2) α′=arg min||α||0s.t.DICα=y

Here ${\left\|\alpha \right\|}_{0}$ is the number of non-zero coefficients in ***α***. ***α'*** is the approximate value of ***α***. Orthogonal matching pursuit (OMP) is used to solve the L_0_ problem. Then we can get approximately reconstructed signal via Equation (3):

(3) $$y^{\prime }=DIC\alpha^{\prime }$$

#### 4.3.2. Dictionary

From the sparse representation model mentioned above, the dictionary plays an important role in the process of sparse decomposition and signal reconstruction. To match each state, *w* features are randomly selected from each **F***_ijk_* to stack into dictionary **DIC***_ijk_*. In order to avoid dictionary redundancy, KSVD is used in our paper to learn an over-completed, but small, dictionary. Two-thirds of each **F***_ijk_* are used to train and update the atoms of **DIC***_ijk_*. Then we can get an excellent sparse decomposition performance at the corresponding state.

#### 4.3.3. Sparse Representation Classification

To extend the application of sparse representation, sparse representation classification was first proposed by Wright *et al.* for face recognition in [41]. In the case of vigilance detection, we will introduce a SRC model as follows. Here, **DIC**_a_ = [**d**_a1_,… ,**d**_aw_] represents the dictionary of alert state and **DIC**_d_ = [**d**_d1_,… ,**d**_dw_] represents the dictionary of alert or drowsy. We form these two sub-dictionaries as **DIC** = [**DIC**_a_ **DIC**_d_]. In this way, a test sample y can be represented as Equation (4):

(4) $$y=DIC\alpha =\sum d_{a1}\alpha_{a1}+\cdots +d_{aw}\alpha_{aw}+d_{d1}\alpha_{d1}+\cdots +d_{dw}\alpha_{dw}$$

By combining Equations (2) and (3), we can get the sparse coefficient $\alpha^{\prime }=[\alpha_{a1},\ldots ,\alpha_{aw},\alpha_{d1},\ldots ,\alpha_{dw}]$

So, $\alpha_{a}^{\prime }=[\alpha_{a1},\ldots ,\alpha_{aw},0,\ldots ,0]$ can be regarded as the sparse coefficients under **DIC**_a_, and $\alpha_{d}^{\prime }=[0,\ldots ,0,\alpha_{d1},\ldots ,\alpha_{dw}]$ can be regarded as the sparse coefficients under **DIC**_d_. The class of **y** is calculated by Equation (5) and is determined by the minimal residual of **y** and its reconstructed signal $y^{\prime }$ under coefficients of $\alpha_{a}$ and $\alpha_{d}$:

(5) $$class(y)={\mathrm{argmin}}_{x}residual_{x}{\left\|y-DIC\alpha_{x}^{\prime }\right\|}_{2}$$

where x = [*a*, *d*].

If the driver is alert, the non-zero sparse coefficients will concentrate on these sections corresponding with **DIC**_a_. Then the residual in the condition of $\alpha_{a}$ is smaller than in the condition of $\alpha_{d}$ in theory. We can determine what state the driver is in. After that, our proposed vehicle speed control strategy receives the driver’s vigilance level to determine when and how we should decelerate our vehicle speed to ensure safety.

## 5. Vehicle Speed Control Strategy

**Figure 6:**
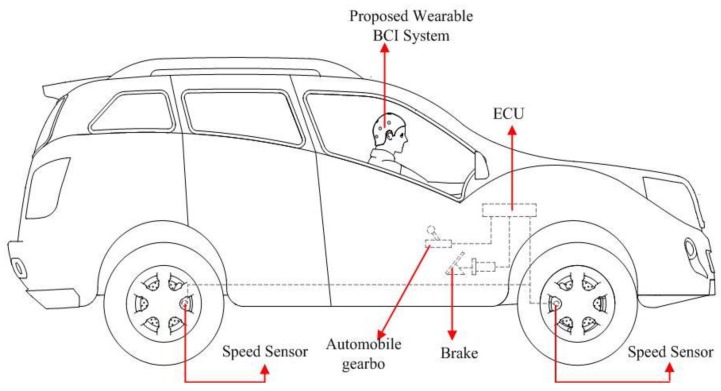
Prototype of proposed vehicle speed control strategy.

In this section, a novel safety strategy of vehicle speed control, which controls the electronic throttle opening and automatic braking after driver fatigue detection using the above method, is presented to avoid serious collisions and traffic accidents. As shown in Figure 6, if the driver’s drowsiness is confirmed, the algorithm gives different operating commands, including deceleration and braking. The ECU receives these operation commands and controls the vehicle to avoid traffic accidents. Auto-braking and deceleration, a vehicle deceleration algorithm based on the car following security distance model, and the relationship between vehicle speed is presented in this section.

Different operations are used to prevent traffic accidents from happening in different circumstances. For example, immediate braking is used under emergency situations. In those cases, the accelerator is cut off and the braking system begins to work automatically. In other words, at that moment, an acceleration pedal operation will be replaced by an automatic braking operation, as in the right block of Figure 1. Speed control is used in the whole driver vigilance detection process to get an appropriate speed of the vehilce.

### 5.1. Vehicle Deceleration Algorithm

**Figure 7:**
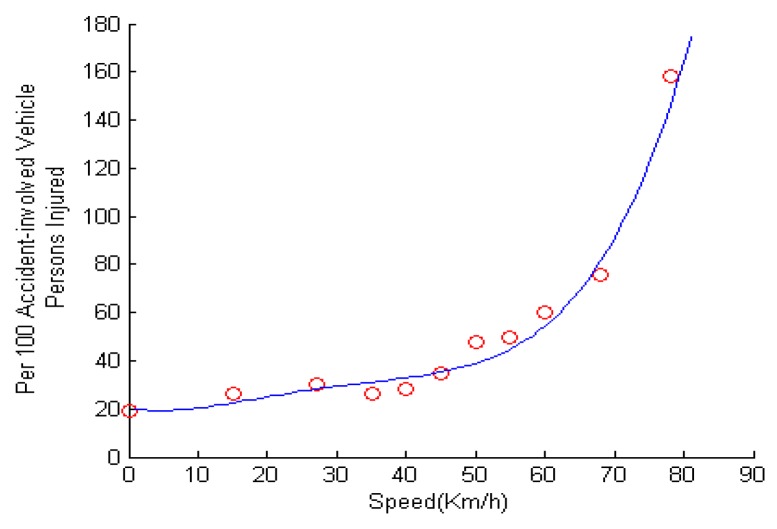
Relationship between accidental risk and speed.

In this subsection, we analyze the vehicle deceleration algorithm. Due to the danger of sudden deceleration, the first and most important part in our vehicle speed control strategy is the safety of deceleration. However, this poses two challenges, one of which is how we can determine the speed difference (ΔV) between initial speed and end speed after deceleration. Another is to choose a proper accelerometer to avoid rear-end collisions in the course of deceleration. From the theory in [42], we have the relationship between accidental risk and speed, as shown in Figure 7. We find that the accidents occurring in the case of moderate or high speed driving are more severe than in the case of low speed.

**Figure 8:**
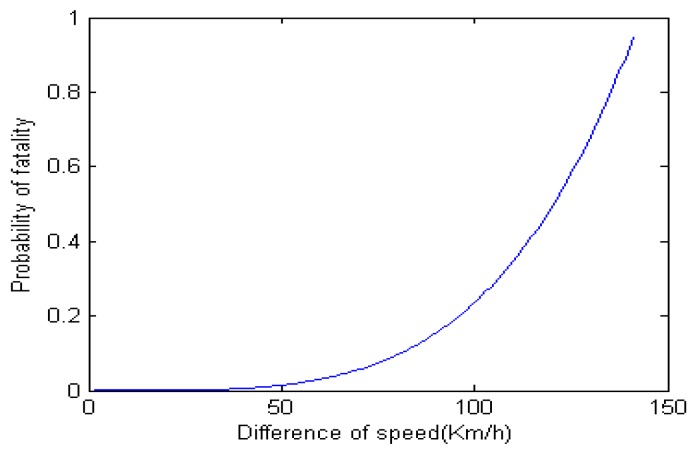
Relationship between the speed difference and probability of fatality.

Another study from Joksch [43] indicates that the relationship between the speed difference and probability of fatality in Figure 8. This means that we should keep the speed difference with other vehicles less than 20 km/h as much as possible. Thus, it is proper to choose ΔV = 20 km/h to reduce the speed of vehicle in the case of drowsiness. Additionally, a car-following safety distance model is adopted to calculate an accelerometer to avoid the rear-end collision in the course of deceleration. Some parameters are defined as follows:

- *t_1_*: The driver reaction and brake coordination time of following vehicle;
- *t_2_*: The acceleration increase time of following vehicle;
- *t_3_*: The uniform deceleration time of following vehicle;
- t*_following_*: The total time of following vehicle deceleration;
- *v_21_*: The initial speed of following vehicle;
- *v_22_*: The final velocity of following vehicle calculated by *v_12_* = *v_21_* − 20 Km/h;
- *s_1_*: The distance traveled by the following vehicle during *t*1;
- *s_2_*: The distance traveled by the following vehicle during *t*2;
- *s_3_*: The distance traveled by the following vehicle during *t*3;
- *s_following_*: The distance traveled by the following vehicle during the whole process;
- *a_m_*: The maximum accelerometer of the following vehicle;
- *v_11_*: The initial speed of the front vehicle;
- *v_12_*: The final velocity of front vehicle calculated by *v_12_* = *v_21_* − 20 Km/h;
- *a_f_* : The accelerometer of the front vehicle;
- D*_min_*: The minimum safety distance of the front-following vehicle after deceleration;
- *L*: The needed distance of front-following vehicle before deceleration; and
- s*_front_*: The distance traveled by the front vehicle during the whole process.

Generally, under the assumption that the following driver immediately decelerates, the deceleration process of the following vehicle can be divided into three stages: (1) driver’s reaction and brake coordination stage; (2) acceleration increase stage; and (3) uniform deceleration stage.

**Driver’s reaction and brake coordination stage (*t_1_*):** the following driver gets the deceleration information of the front car and then controls the vehicle. The travel distance *s_1_* during *t_1_* can be represented as Equation (6):

*s_1_*=*t_1_v_21_* (6)

**Acceleration increase stage (*t_2_*):** the acceleration of the following car increases from zero to *a_m_*. The travel distance *s_2_* during *t_2_* can be calculated by Equation (7):

(7) $$s_{2}=v_{21}t_{2}+\frac{a_{m}}{6}{t_{2}}^{2}$$

**Uniform deceleration stage (*t_3_*):** the speed slows down at an accelerometer of am to *v_e_*. The travel distance *s_3_* and *t_3_* can be calculated by Equations (8) and (9):

(8) $$s_{3}=-\frac{1}{2a_{m}}(v_{21}^{2}+\frac{a_{m}^{2}}{4}t_{2}^{2}+v_{21}a_{m}t_{2})$$

(9) $$t_{3}=\frac{2(v_{22-}-v_{21})-a_{m}t_{2}}{2a_{m}}$$

Therefore, we can get *s_following_* and *t_following_* using Equations (10) and (11):

(10) $$s_{following}=s_{1}+s_{2}+s_{3}=v_{21}(t_{1}+\frac{t_{2}}{2})+\frac{v_{22}^{2}-v_{21}^{2}}{2a_{m}}+\frac{a_{m}}{24}t_{2}^{2}\approx v_{21}(t_{1}+\frac{t_{2}}{2})+\frac{v_{22}^{2}-v_{21}^{2}}{2a_{m}}$$

(11) $$t_{following}=t_{1}\;+t_{2}+t_{3}=t_{1}\;+t_{2}+\frac{2(v_{22-}-v_{21})-a_{m}t_{2}}{2a_{m}}$$

When the driver is detected to be in danger case, we let the front car decelerate from *v_11_* to *v_12_* during *t_following_*, and we can get the accelerometer of *a_f_* from Equation (12) and *s_front_* from Equation (13):

(12) $$a_{f}=\frac{v_{12}-v_{11}}{t_{following}}$$

(13) $$s_{front}=\frac{v_{12}^{2}-v_{11}^{2}}{2a_{f}}$$

By combination of Equations (11) and (12), *a_f_* is simplified as Equation (14):.

(14) $$a_{f}=\frac{2a_{m}(v_{21}-v_{11}-20)}{a_{m}(2t_{1}+t_{2})+2(v_{22}-v_{21})}$$

By combination of Equations (13) and (14), *s_front_* is simplified as Equation (15):

(15) $$s_{front}=\frac{1}{4a_{m}}(v_{21}-v_{11}-20)(a_{m}(2t_{1}+t_{2})+2(v_{22}-v_{21}))$$

**Figure 9:**
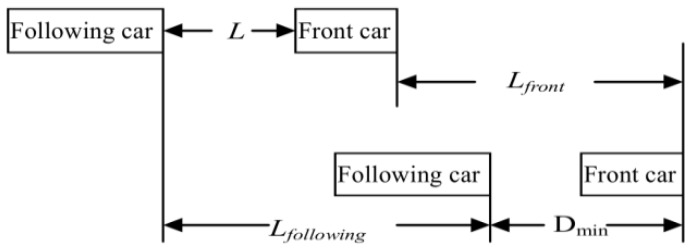
Car following safety model.

As shown in Figure 9, *L* can be calculated through Equation (16) by combining Equations (10) and (15):

(16) $$L=D_{\min}+s_{following}-s_{front}=D_{\min}+v_{21}(t_{1}+\frac{t_{2}}{2})+\frac{v_{22}^{2}-v_{21}^{2}}{2a_{m}}-\frac{v_{12}^{2}-v_{11}^{2}}{2a_{f}}$$

If the actual distance *L_a_* of front-following vehicle is longer than the need distance *L*, it is safe.

Table 1 shows the range of some parameters used in this paper. Furthermore，a binocular vision system is used to obtain *L_a_*. In the binocular vision system, the binocular-cameras is a homemade device at our lab. The binocular digital cameras have 320 × 240 resolution, and our algorithm is implemented and coded with C++ and the OpenCV library on a laptop computer equipped with an Intel i5 2.5 GHz CPU and 4 GB RAM. The parameter *v_21_* can also be obtained by using the binocular vision in our experimental system. In this paper, *L_1_* and *L_2_* represent the distance of front-following car. They are measured with time internal of 0.5 s. The parameter *v_21_* can be represented as Equation (17):

(17) $$v_{21}=v_{11}+2(L_{2}-L_{1})$$

### 5.2. Vehicle Speed Control Strategy

The vehicle speed control strategy is used to determine how we should control the speed of the vehicle after driver vigilance detection. Firstly, we define three situations as follows:

- **Situation 1:** If the driver is detected to be drowsy for a constant *n* s, the driver is regarded as drowsy and the “deceleration” command is sent to the ECU. In this paper, the parameter *n* is variable according to different conditions, and we set *n* at 3 s in our experimental and simulation system.
- **Situation 2:** After Situation 1, if the driver is detected to be not completely alert in next *u* + *z* s (*z* is the time after *u*. *u* ≤ *k*, *z* < *m*, and *u* + *z* ≥ *k*), the driver is regarded as very drowsy and the “braking” command is sent to the ECU in Figure 6.
- **Situation 3:** After Situation 1, if the driver is detected to be not completely alert in the next *u* s but alert in *m* s after *u* (*u* ≤ *k*), the driver is regarded as awake and the “releasing maximum speed limit” command is sent to ECU.

*u* and *z* are time variables for drowsy time and waking time, which are used to record the time that the driver is detected as being in the alert or drowsy state. The parameters *k* and *m* are thresholds. The variable *k* is the time used to wake up driver when the driver is drowsy, and the variable *m* is the minimum time for the driver to be woken up. If the waking up time is less than *m*, the driver has not been awoken.

**Figure 10:**
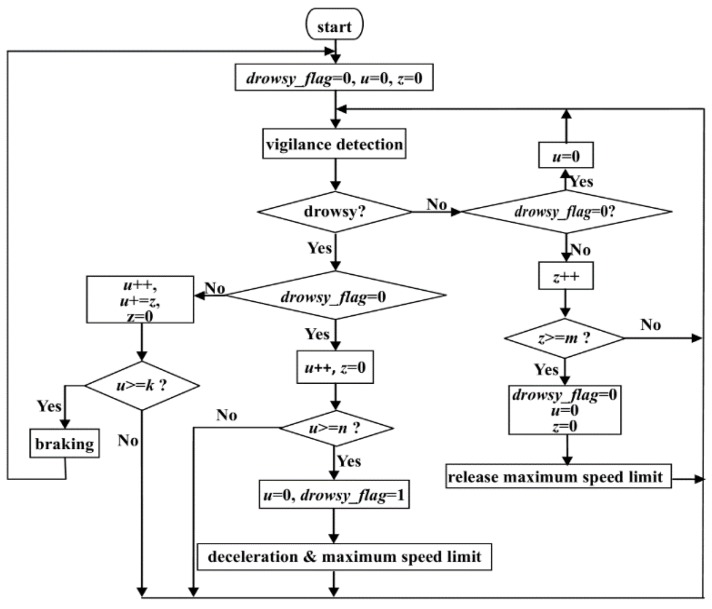
Flowchart of our vehicle speed control strategy.

As shown in Figure 10, we introduce a novel vehicle speed control strategy as follows:

When Situation 1 occurs, the ECU automatically operates the binocular cameras in our experimental system to acquire the *v_21_* and *L_a_*. If *L_a_* > *L*, the ECU controls vehicle deceleration to *v_22_* Km/h with the acceleration of *a_f_* , and then keeps the maximum speed limit at *v_22_* km/h. At the same time, the ECU controls the vehicle horn to wake the driver up. If Situation 2 happens, it indicates that the driver has not been woken up by the horn during time *k* or the waking up time is less than *m*. The ECU accepts the “braking” command and operates the binocular cameras to acquire the *v_21_* and *L_a_*. If *L_a_* > *L*, the ECU controls vehicle braking slowly with the acceleration of *a_f_* for avoiding traffic accidents because of driver deep drowsiness.

Otherwise, if Situation 3 occurs, it indicates that the driver has been woken up by the horn during time *k* and keeps awake at least for time *m*. In this condition, the ECU releases the maximum speed limit. Then, the vehicle is controlled by the driver normally. Based on the above vehicle speed control strategy, the system can control electronic throttle opening and automatic braking to reduce accident rate for avoiding drowsy driving. This makes driving safer and more reliable.

The detail of the flowchart of our vehicle speed control strategy is shown in Figure 10. The variable of *drowsy_flag* is a flag of whether Situation 1 occurs.

**Figure 11:**
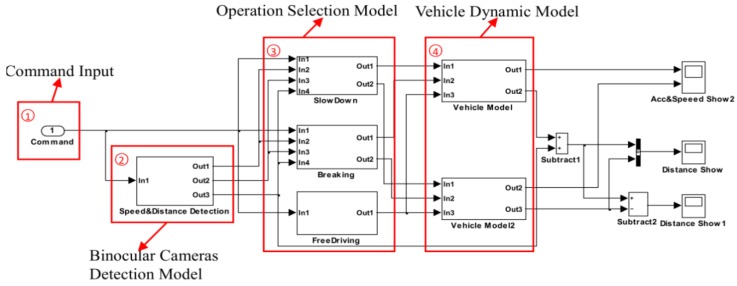
Vehicle speed control model based on the vehicle dynamic model.

To further illustrate flowchart of our vehicle speed control strategy, the vehicle speed control model based on the vehicle dynamic is shown in Figure 11 and executed in our simulation and experimental system. The red box 1 in Figure 11 is the operation command input port used to receive the command, which is determined by our vehicle speed control strategy. The binocular camera detection model which was coded with C++ and OpenCV library in red box 2, is used to measure *L_a_* and *v_21_* of following car when operation commands is received. The operation selection model shown in red box 3 judges which commands is received and exports control information to vehicle dynamic model in red box 4. The vehicle dynamic model performs operation to meet the received command for safer driving.

## 6. System Simulation and Validation

**Figure 12:**
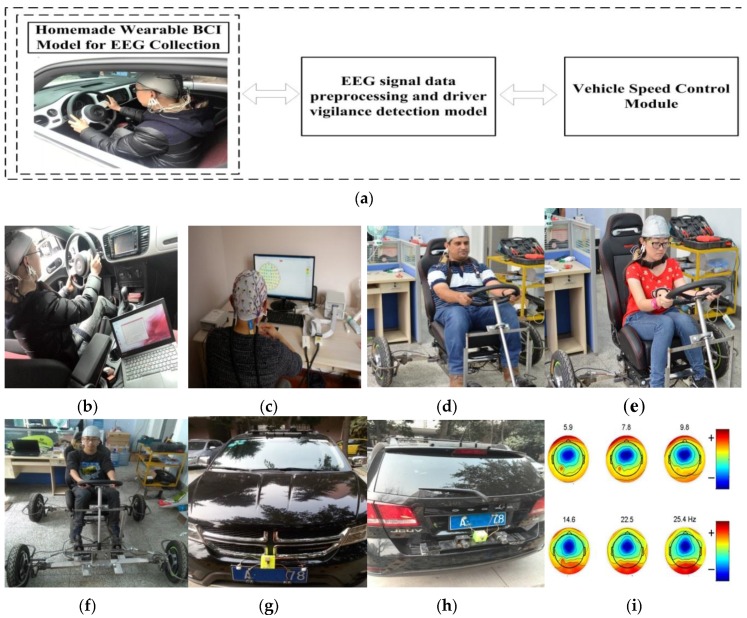
Experimental environment. (**a**) Experimental prototype; (**b**) EEG collection using the homemade wearable BCI system; (**c**) Complementary experiment using BP equipment; (**d**) EEG collection experiment; (**e**) EEG collection experiment; (**f**) EEG collection experiment; (**g**) The test vehicle configuration; (**h**) The test vehicle configuration; (**i**) The driver vigilance detection experiment.

We have implemented an experimental environment to evaluate the proposed system’s performance. The experimental environment consists of three parts as shown in Figure 12a. The first part is a homemade wearable BCI model for EEG collection. The second part is EEG signal data preprocessing and the driver vigilance detection model. The last part is the vehicle speed control module that controls the electronic throttle opening and automatic braking to reduce the accident rate.

### 6.1. Experiment of Homemade Wearable BCI Model for EEG Collection

As shown in Figure 12, we use the real driving environment and complementary simulation in the laboratory to our proposed method. Figure 12d–f illustrate the EEG collection and vigilance detection experiments of our system simulation and validation. In order to confirm the validation of our homemade wearable EEG BCI system, complementary experiment and simulation using BP equipment are shown in Figure 12c. BP is a 64-channel EEG commercial unit that the psychological research and counseling center of Southwest Jiaotong University bought from Germany. Our algorithm is implemented on a laptop computer equipped with an Intel i3 1.9 GHz CPU and 4 GB RAM. This system has been field tested on our homemade experimental vehicle in Figure 12d–f, and been done simulation on a DODGE SUV vehicle as shown in Figure 12g,h. The experimental vehicle is a DODGE SUV equipped with binocular cameras and other sensors to detect the safety distance for the vehicle deceleration algorithm and vehicle speed control. Figure 12i shows the sample image frame from the experiment for driver vigilance detection.

In our experiment, ten qualified drivers, having no neurological diseases, wore the wireless wearable BCI system in Figure 12d–f to collect the EEG signal in Table 2. The experiments of EEG collection and diver vigilance detection are the actual data and field testing. The homemade experimental vehicle is designed to test driver vigilance for avoiding traffic risk of actual fatigue driving tests. The experimental conditions are set as follows:

- **Condition 1:** (1) sleep deprivation; (2) test time is the next day between 4 a.m and 6 a.m;
- **Condition 2:** (1) having a normal night sleep; (2) test time is the next day between 9 a.m and 11 a.m.

**Figure 13:**
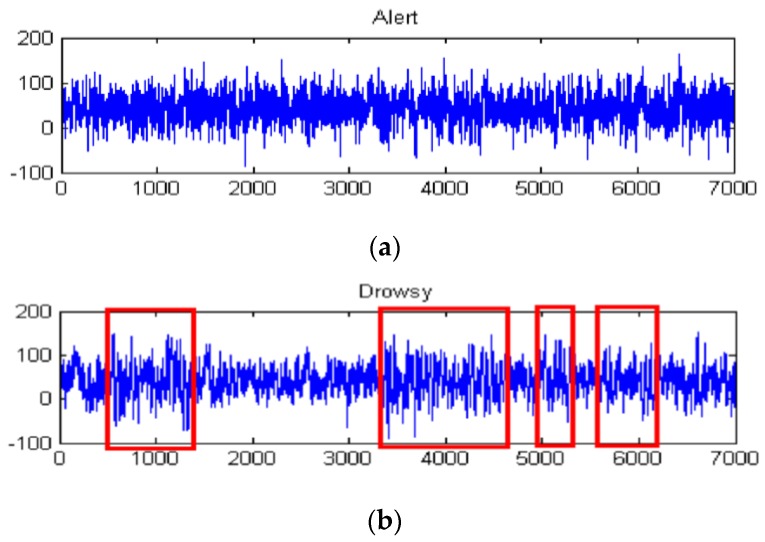
Original EEG signal collected from our proposed equipment. (**a**) Alert signal; (**b**) Drowsy signal.

During the whole experiment process, the investigators observe and record the subjects’ physical behaviors, yawns, and the inclination of head, as a drowsiness index in the next study. Finally, each **Set**_ijk_ composed of 20 min sessions is collected from both nits. **Set**_BP_ contains 63 channels EEG/EOG signals with a sampling frequency of 1000 Hz. **Set**_HBS_ includes two EEG channels, O1 and O2, with a sampling frequency of 512 Hz. Figure 13 shows the original EEG signal collected from a 24 year old experimental driver. Figure 13a is the alert signal, Figure 13b is the drowsy signal. The signal in the red box is alpha activity bursting in the drowsy state. Taking into account the risk of actual fatigue driving tests, simulation in the scenario is used to test driver vigilance for avoiding traffic risks of actual fatigue driving tests. This is the limitation of the presented simulation and we will construct an optimization experiment in future research.

### 6.2. Experimental Results for EEG Preprocessing and Driver Vigilance Detection Model

#### 6.2.1. Preprocessing Experiment

**Figure 14:**
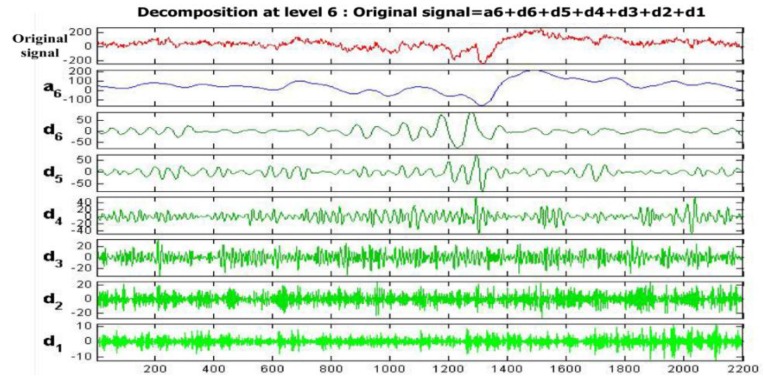
Original signal and its decomposition signal at each level.

In this subsection, the goal of preprocessing is to improve the original data quality. The original EEG signal selected from **Set**_HBS_ is used in this experiment. Figure 14 shows the original signal and its decomposition signal at each level of one experimental driver. Figure 14 shows the original signal. The six level decomposition signal is uses db5. In Figure 14, the reconstructed signal a_6_ is the reconstructed signal of approximate coefficients at level 6. The reconstructed signals of d_6_ to d_1_ are the reconstructed signals of detail coefficients at levels 6 to 1, as shown in Figure 14. The frequency range of each reconstructed signal is shown in Figure 4. Since the hardware filter of our homemade equipment is 3–100 Hz, the signal of 0–3 Hz can be regarded as noise. In addition, the frequency range of the EEG is between 0.5 and 50 Hz.

**Figure 15:**
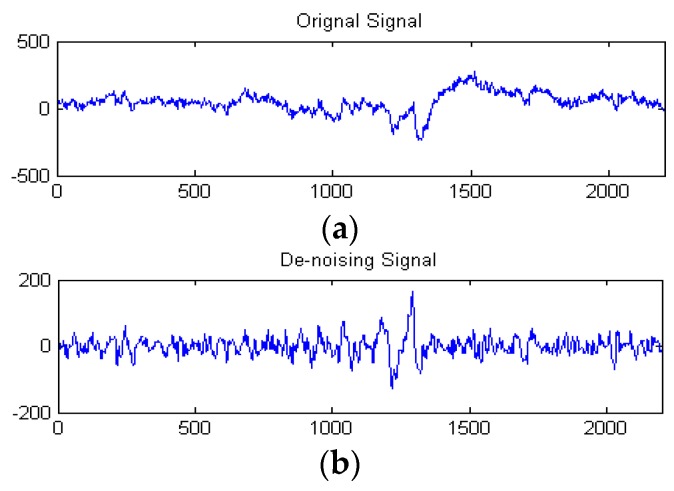
(**a**) Original signal s; (**b**) De-noising signal.

**Figure 16:**
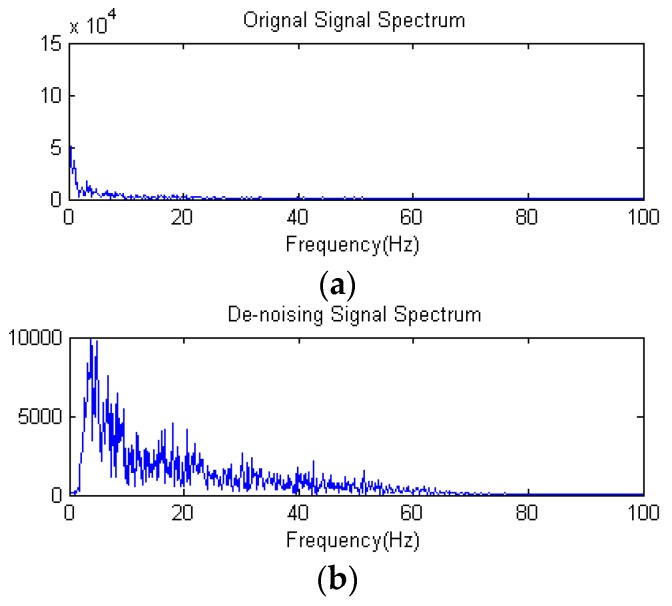
(**a**) Original signal spectrum; (**b**) De-noising signal spectrum.

**Figure 17:**
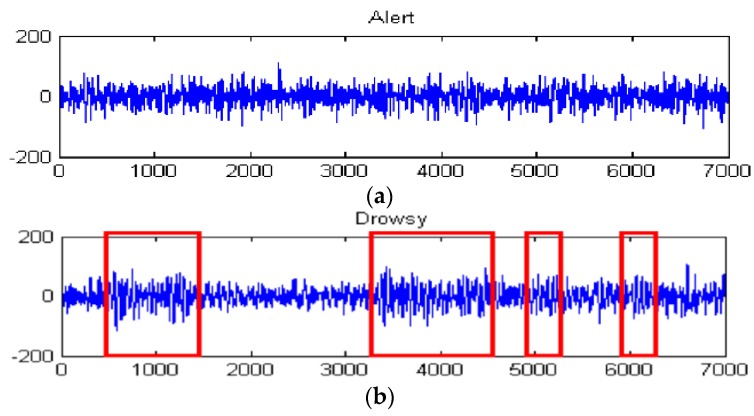
De-noising signal of signal shown in Figure 13. (**a**) Alert signal; (**b**) Drowsy signal.

The high-frequency signal (>50 Hz) can be regarded as interference. Therefore, by extracting the decomposition signal of d_3_ (32–64 Hz), d_4_ (16–32 Hz), d_5_ (8–16 Hz), d_6_ (4–8 Hz), we can get the useful EEG signal and remove some low- and high-frequency interference. Figure 15a and Figure 15b show the original signal and de-noising signal of the above experimental driver. It gives good performance of de-noising. Figure 16 shows the spectrum of them. As shown in Figure 16a, the low-frequency noise is too large to overwhelm EEG signal. In Figure 16b, the frequency of EEG is retained well and some of low frequency and high frequency noise are removed. Furthermore, we implement the de-noising algorithm in signals shown in Figure 13 to verify the de-noising performance in two states. Figure 17 shows a de-noising signal of alert and drowsy states We can see the alpha band is more obvious from other bands in the drowsy state.

#### 6.2.2. Feature Extraction Experiment

**Figure 18:**
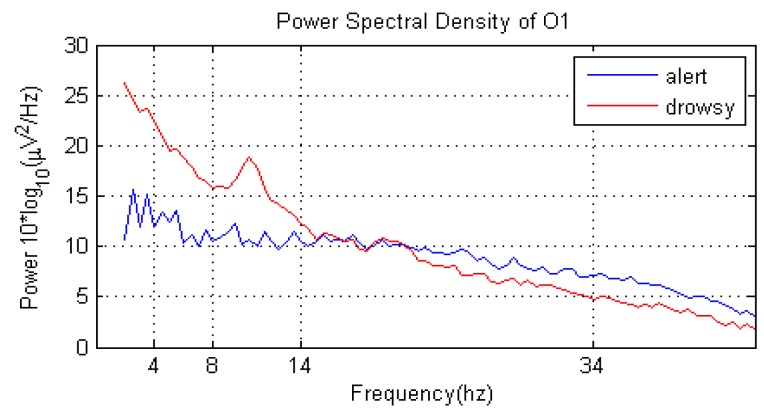
PSD of the whole testing data.

**Figure 19:**
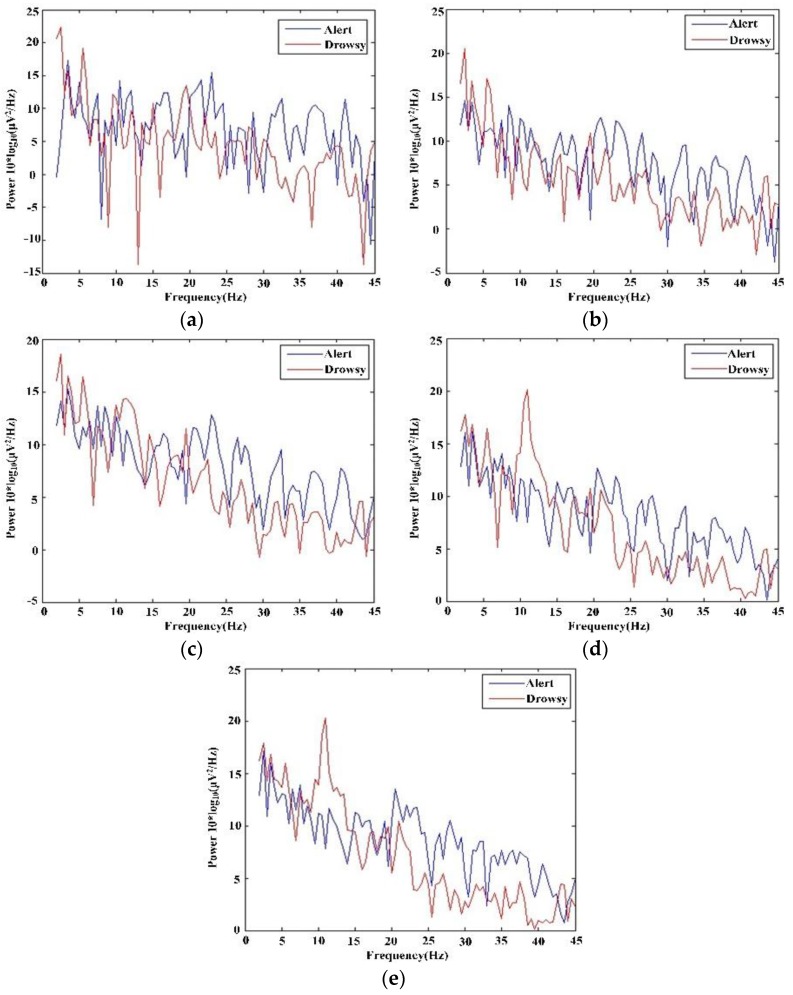
PSD of *t-*th s when s adopt (**a**) 0; (**b**) 2; (**c**) 4; (**d**) 6; and (**e**) 8.

Under the assumption of the driver’s state at time *t* remains the same state with the previous *b* and *s*, the feature of the EEG signal at time *t* is calculated as the average PSD of previous *b* s and *t* s. The PSD is calculated using FFT. Generally, the more data is used in feature extraction, the more precise the feature . Meanwhile, this means that more time is spent. Figure 18 shows the PSD of the whole testing data. Figure 19 shows the PSD of *t-*th s under different *b* (*b* = [0, 2, 4, 6, 8]). The blue line represents the PSD of driver alert state and red line represents driver drowsy state. When *b* = 0 or *b* = 2 or *b* = 4, the discrimination of whether the driver is alert or drowsy is not distinct. When *s* increases to *b* = 6 or *b* = 8, the discrimination is much obvious. As shown in Figure 18, the power of theta and alpha rhythms in the driver drowsy state is greater than in the driver alert state. The power of the beta rhythm in the driver drowsy state is smaller than in the driver alert state. In addition, with the increase of *b*, the tendency of PSDs in Figure 19 gradually meet the PSD tendency shown in Figure 18. To estimate the running time of different *b*, the average time of 10,000 runs of the PSD algorithm is calculated, as shown in Table 3. The running time increases with the increase of *b*. That is, we should find a trade-off between the precision and time.

#### 6.3.3. Classification Experiment

Orthogonal matching pursuit (OMP) is used to solve the L_0_ problem. Let *q* represent the number of atoms in each linear combination. The greater the numbers of atoms, the easier the accuracy of the signal can be reconstructed. The class of the driver vigilance level is determined by Equation (5). The classification accuracy rate of our proposed algorithm is calculated by Equation (18):

(18) $$accuracy\;rate=\frac{No.\;of\;correctlly\;detection}{No.\;of\;total\;detection}$$

Table 4 and Table 5 show the classification rate of O1 and O2 from **Set**_HBS_. Driver 1, Driver 2, Driver 3, and Driver 4 are the number of different persons of ten qualified drivers. We can see that with the increase of *b*, the classification rate increases corresponding with the results discussed in the subsection of the feature extraction experiment. Although other algorithms have been used previously for the same task, we wanted to try to create a novel algorithm for driver vigilance detection using sparse representation classification and KSVD. In our lab, we have a strong research basis on the sparse representation classification algorithm, and we find that sparse representation classification combined with KSVD has good performance in driver vigilance classification. Thus, in this paper, we firstly introduced the proposed method to driver vigilance detection. At the same time, for our vehicle active safety model, we think the classification accuracy rate is suitable to vehicle speed control. As in the red box of Table 5, when *b* ≥ 7, our equipment gives an excellent classification efficiency which is up to around 93%, and it is match the result of feature extraction experiment (the PSD are obvious difference as Figure 19d,e). These results indicate that the proposed method has good performance in driver vigilance detection. The classification rates of **Set**_BP_, O1 and **Set**_BP_, O2 are shown in Table 6 and Table 7. In our experiment, the experiments demonstrate the validation of the driver vigilance detection algorithm based on using EEG and sparse representation.

#### 6.3.4. Vehicle Speed Control Experiment

To simplify the validity of our vehicle deceleration model, we assume that:

1. *v_11_* = 95 km/h, *v_21_* = 100 km/h, *L_a_* = 10.5 m (The distance of *L* is calculated through Equation (16) and equal to 10.39 m, so condition of *L* < *L_a_* is satisfied);
2. When drowsiness is detected, the front car decelerates and an accelerometer of *a_f_* is calculated by Equation (16);
3. The following car catches the information of the front car and immediately decelerates.

**Figure 20:**
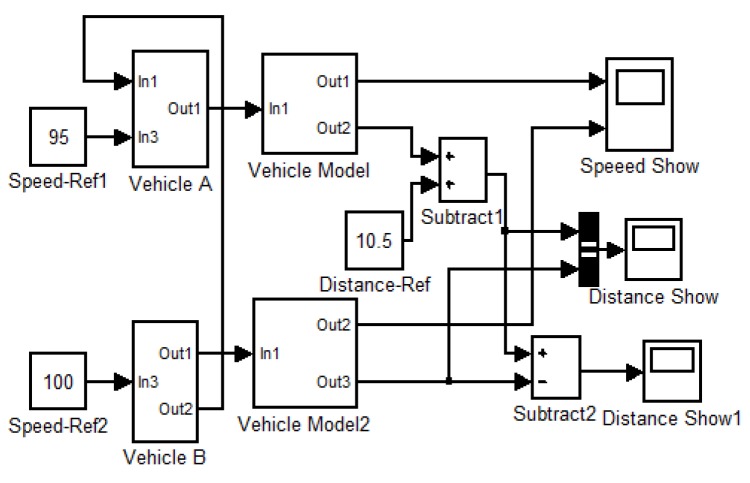
Vehicle following model.

**Figure 21:**
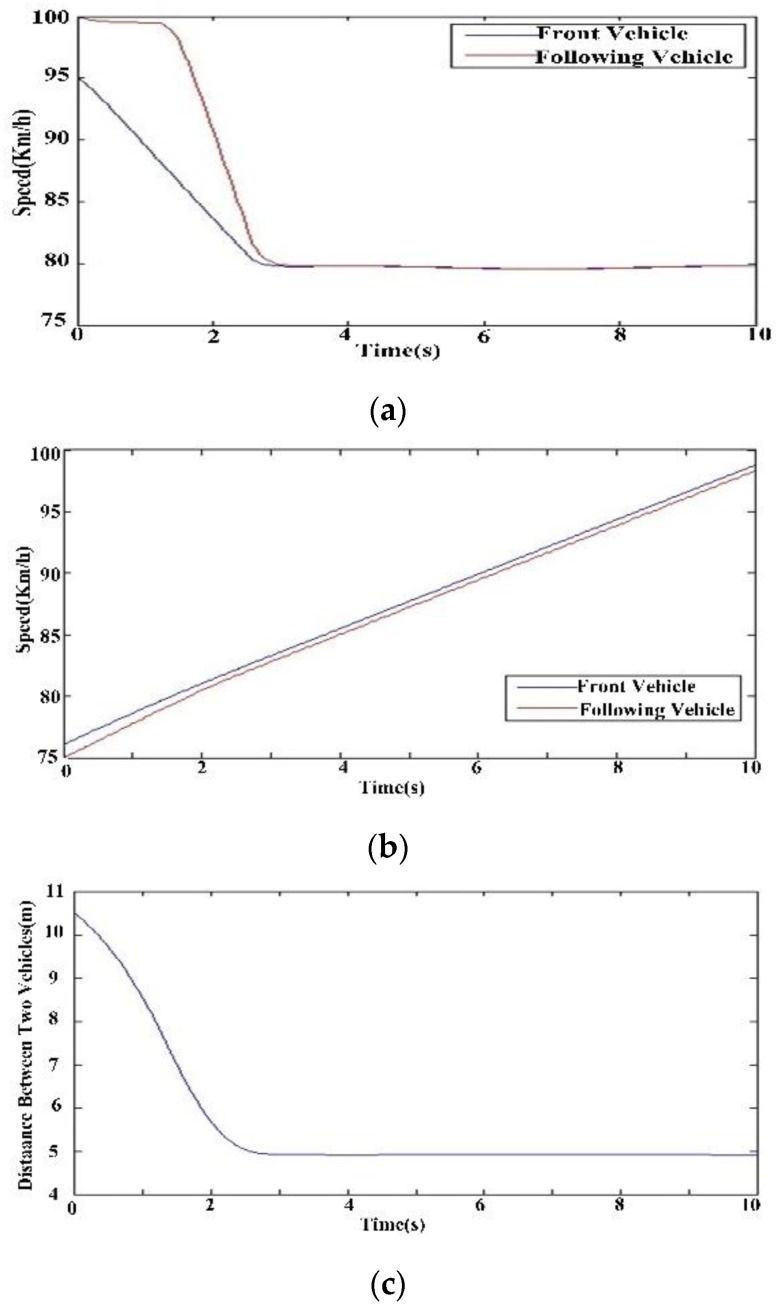
Changes of two cars in the course of deceleration. (**a**) Change of speed; (**b**) Change of distance; (**c**) Change of distance.

Figure 20 shows the vehicle following model based on the dynamics using *MATLAB*/*simulink*. The simulation results in Figure 21 shows in the course of deceleration, the speeds of two cars slow down to 80 km/h in Figure 21a, and the distance between the two cars decreases from 10.5 m to 5 m in Figure 21b,c. The deceleration model is valid for the avoidance of a rear-end collision when driver vigilance is detected.

**Figure 22:**
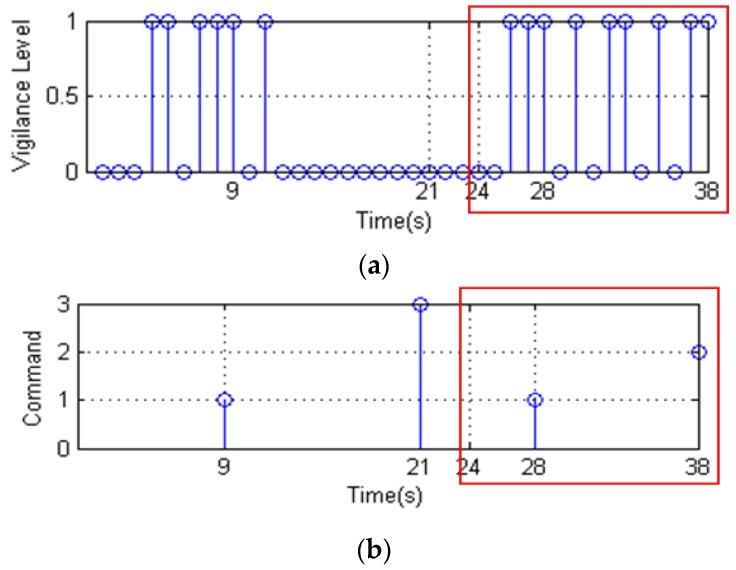
Result of vehicle speed control strategy. (**a**) Vigilance level list; (**b**) Command list.

We assume that the vigilance level list is detected as Figure 22a. The “0” represents alert and the “1” represents drowsiness. To facilitate simulation, we set *n* = 3 s, *k* = 10 s, and *m* = 10 s. The command list is shown in Figure 22b. Where, “1”, “2”, and “3” represent deceleration, braking, and releasing the maximum speed limit, respectively. As shown in Figure 22a, the driver is detected to be drowsy for 3 s from the 7th s to the 9th s It meets the criteria for Situation 1, and the deceleration command occurs in the 9th second. Similarly, at the 21st, 28th, and 38th s, in turn, it meets the criteria for Situation 3, 1, and 2, and the commands are releasing maximum speed limit, decelerating, and braking.

**Figure 23:**
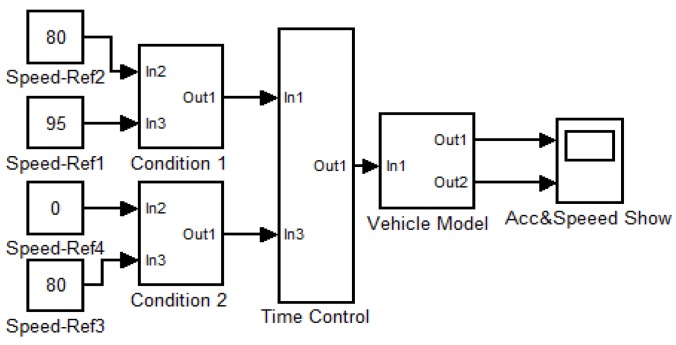
Simplified vehicle speed control model.

**Figure 24:**
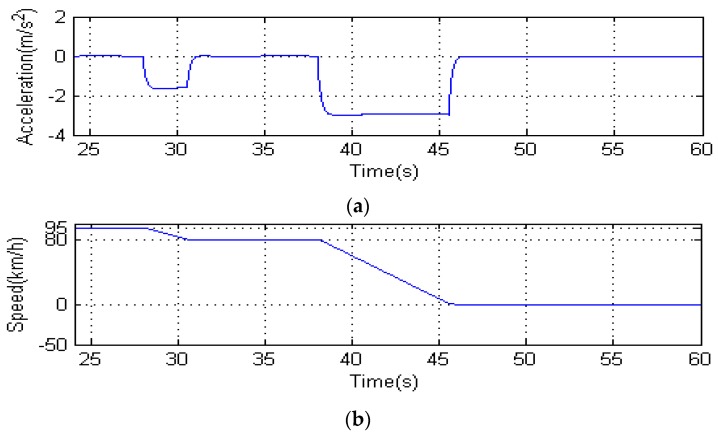
results of vehicle speed control. (**a**) shows the change acceleration; (**b**) shows the change of speed.

Since we cannot predict the behavior of the driver after the speed limit is released, we cannot forecast the behavior of the vehicle. We only verify the commands during 24 s to 38 s in the red box of Figure 22 below the speed limit. Figure 23 shows a simplified model of vehicle speed control, which is designed for the case of red box in Figure 22b. In this model, we assume that *v_11_* = 95 km/h, *v_21_* = 100 km/h, *L* < *L_a_* at time 24 s Figure 24a,b show the change of acceleration and speed. At the 28th s, the ECU receives the deceleration command and controls the vehicle deceleration to 80 km/h. At the 38th s, the ECU receives the braking command and controls the vehicle braking slowly. The experiment and simulation show the validity of our vehicle speed control strategy when the driver is deeply drowsy.

## 7. Conclusions

In this paper, we have presented a vehicle active safety model for drowsy driving based on driver vigilance detection using wearable EEG and sparse representation. The methods have three steps, namely, wearable EEG collection, vigilance detection, and vehicle speed control strategy. In the first step, a homemade low-cost, comfortable, wearable BCI system with eight channels is designed for collecting the driver’s EEG signal. In the vigilance detection step, wavelet analysis is used for de-nosing and FFT is introduced to calculate the PSD for extracting EEG features. Next, sparse representation classification combined with k-singular value decomposition (KSVD) is firstly introduced in PSD to estimate the driver’s vigilance level. In the last step, a novel safety strategy of vehicle speed control, which controls the electronic throttle opening and automatic braking after driver fatigue detection using the above method is presented to avoid serious collisions and traffic accidents. The final experimental results show the validity of our method under the simulated and realistic conditions. From both theoretical analysis and practical experiments, it shows that the proposed system has not only good performance of EEG collection and vigilance detection, but also effective controls of vehicle speed. The result of the experiment shows that the proposed homemade wearable BCI system is accurate for EEG collection. The sparse representation is an effective method for vigilance detection. At the same time, the vehicle speed control strategy is effective when the driver is drowsy. The vehicle deceleration algorithm is effective in preventing collisions in the course of speed control.

## Figures and Tables

**Table 1:** The range of parameters and value used in this paper.

	*t*_1_	*t*_2_	*a*_m_	*D*_min_
Range	0.5~1.5	0.2/0.7	0~6.0	2~5
Value	1.2	0.2	4.5	5

**Table 2:** Ten Drivers Served in The Experiment.

Driver Sum	Subject	Number	Age
10	Male	7	26
			26
			38
			42
			23
			23
			24
	Female	3	24
			24
			25

**Table 3:** Running time of different *b*.

*b*(s)	0	2	4	6	8
Time(s)	0.0006	0.0015	0.0020	0.0022	0.0025

**Table 4:** Classification accuracy rate (%) of **Set**_HBS,O1_.

b(s)	Driver1	Driver2	Driver3	Driver4
0	74.72	56.85	72.72	46.42
1	74.64	77.24	73.14	62.58
2	78.57	61.11	76.43	73.18
3	81.38	61.54	75.14	67.15
4	85.63	78.87	84.30	72.79
5	85.59	52.48	88.30	71.11
6	90.75	72.85	88.23	82.83
7	91.59	87.05	97.04	78.94
8	93.02	83.33	82.73	84.84

**Table 5:** Classification accuracy rate (%) of **Set**_HBS,O2_.

b(s)	Driver1	Driver2	Driver3	Driver4
0	74.42	47.65	59.88	72.72
1	78.92	66.14	76.13	77.77
2	81.43	69.04	78.85	79.60
3	87.97	92.80	79.31	85.43
4	91.38	62.90	76.30	85.33
5	92.22	71.54	89.53	82.55
6	94.80	97.54	87.71	82.43
7	95.65	95.04	96.47	93.87
8	95.35	96.66	98.22	94.55

**Table 6:** Classification accuracy rate (%) of **Set**_BP,O1_.

b(s)	Driver5	Driver6	Driver7	Driver8
0	66.82	64.02	67.76	56.54
1	70.09	69.16	69.16	73.83
2	75.70	74.77	73.83	73.83
3	75.59	79.81	73.24	79.34
4	73.71	80.28	77.93	62.91
5	69.48	67.14	82.63	69.95
6	69.34	90.09	85.38	83.96
7	92.45	91.04	94.34	85.38
8	95.75	99.06	94.34	85.38

**Table 7:** Classification accuracy rate (%) of Set_BP,O2_.

b(s)	Driver5	Driver6	Driver7	Driver8
0	62.15	59.81	64.95	71.03
1	66.36	65.42	69.16	68.69
2	73.36	74.77	76.17	78.50
3	75.59	76.53	80.75	80.28
4	77.46	81.22	77.93	79.81
5	69.95	82.63	80.75	82.63
6	75.00	78.77	82.08	89.15
7	82.55	88.21	91.51	90.09
8	95.28	93.87	97.17	81.13
